# Hastings—Malvern—Leamington

**Published:** 1829-01-01

**Authors:** 


### HASTINGS—MALVERN—LEAMINGTON.

1. ON THE CURATIVE INFLUENCE OF THE SOUTHERN COAST OF ENGLAND; ESPECIALLY THAT OF HASTINGS: WITH OBSERVATIONS ON DISEASES IN WHICH A RESIDENCE ON THE COAST IS MOST BENEFICIAL. By *William Harwood*, M.D. Octavo, pp. 326. Colburn, 1828.
2. A DISSERTATION ON THE NATURE AND PROPERTIES OF THE MALVERN WATER, AND AN ENQUIRY INTO THE CAUSES AND TREATMENT OF SCROFULOUS DISEASES AND CONSUMPTION; TOGETHER WITH SOME REMARKS UPON THE INFLUENCE OF THE TERRESTRIAL RADIATION OF CALORIC UPON LOCAL SALUBRITY. By *William Addison*, Surgeon. Octavo, pp. 192. Callow and Wilson, 1828.
3. A PRACTICAL DISSERTATION ON THE WATERS OF LEAMINGTON SPA; INCLUDING THE HISTORY OF THE SPRINGS, A NEW ANALYSIS OF THEIR GAZEOUS CONTENTS, THE RULES FOR DRINKING THE WATERS, BATHING, DIET OF THE PATIENTS, AND OTHER REGIMEN. By *Charles Loudon*, M.D. Physician at Leamington. 8vo, pp. 92. September, 1828. Longman and Co. London.

THERE is not much in these productions to engage the professional reader. Physicians and surgeons are partial to the localities where they reside and practise; their Essays on such subjects are generally tentative, and are to be received with caution. What we have long known we are naturally favourable to, and when the suggestions of self-interest and the love of fame are in continual operation, the mind imperceptibly acquires a particular bias, while imagination frequently triumphs over judgment. With these reservations, which claim for themselves a general and not a personal application, we think that the works of Dr. Harwood, Dr. Loudon, and of Mr. Addison are respectably executed.

"HASTINGS COAST.

"There is no part of our southern coast whose name has been rendered more familiar to us than this—from its immediate connexion with the Norman conquest, one of the most eventful periods of our history; but as there are many other circumstances relating to its peculiar locality, no less worthy of public attention, I shall mention some of those natural advantages it possesses, which are so obviously calculated to exert an influence beneficial to health; and which, in connexion with others, arising from its proximity to the Metropolis, Brighton, Tonbridge-Wells, and Dover, have justly obtained for it so high a curative reputation. The town is bounded on the north and east by some of the most elevated land in the county of Sussex, and probably the most so on the southern coast of England; the hill of Fairlight, which is about a mile and a half distant, being 541 feet in height. On the west it is screened by a continuous line of hill, rising to an elevation of from 200 to 300 feet; and on the south, the British Channel presents a wide and extensive bay, stretching from Dungeness on the east, to Beachy-head on the west." 16.

"Of all the benefits, however, which the Hastings coast offers to the invalid, there is none more obvious than the choice of situation it affords, adapting it either for summer or winter residence; many of its habitations being placed at an elevation of two or three hundred feet above the level of the sea; consequently, as the temperature of all places is so materially diminished in proportion to their elevation, that in this country, one of 270 feet is allowed to be equal in the difference of its temperature to an entire degree of latitude: and as these more elevated parts of the town of Hastings are moreover visited, during the summer months, by the then prevailing breezes, descending from the surrounding altitudes, these higher parts of the town necessarily receive from them a very diminished temperature, at those periods when coolness is most grateful. While on the other hand, the numerous habitations which are placed on the immediate beach, below the cliffs, being most effectually sheltered, at all seasons, from the more piercing winds, are no less suitably adapted for a winter residence. From hence it follows, that a proper degree of caution should be exercised on the part of invalids, lest by an injudicious choice, between situations so remote from each other in character, a summer or winter residence here, may lose some of its more important advantages."—HARWOOD, 21.

### MALVERN.

"The chain of elevations denominated the MALVERN HILLS, runs in a direction nearly north and south for a distance of almost nine miles, the northernmost extremity, which is about seven miles and a half S. W. of the city of Worcester, being highest and the boldest.

"The summits of the Malvern Hills attain to a heighth of between fourteen and fifteen hundred feet above the level of the sea, and rising up in several places in a pointed or conical manner, in others running along in a narrow undulating ridge, and their sides sloping down in a broken and precipitous descent, render the outline of the whole remarkably picturesque; while the deep ravines, which in numerous places intersect the masses composing the chain, present a grand and romantic appearance when they suddenly burst upon the view." 3.

"Malvern is, perhaps, one of the most healthy and delightful spots in the kingdom, and possesses advantages very rarely indeed to be found combined elsewhere. Nature seems to have unfolded her choicest beauties in the surrounding scenery, and to have collected here every thing that can delight the eye, or engage the imagination. The air has always been justly celebrated for its great purity and invigorating quality; the healthiness of its topographical situation has been acknowledged by all who have resorted to it; whilst its salutary and wholesome water holds out a paramount inducement to those who are suffering from bodily infirmity. It is to an examination of the latter, and to an enquiry into the manner in which it has proved serviceable in scrofulous and other diseases occurring in weak habits, that some of the following pages will be especially appropriated, particularly as it has long been resorted to not only for the cure of these, but also for the alleviation of other important disorders to which mankind are subjected.

"There are two wells here frequented by invalids, one called St. Ann's Well, which is a little distance above the village of Great Malvern, the other, or the Holy Well, is nearly a mile and a half upon the road towards Little Malvern and Ledbury, where are a number of genteel residences and some boarding houses for the accommodation of those, who either for benefit or pleasure, resort to this enchanting spot.

"Both these springs are upon the eastern side of the range, and being situated some distance up the ascent of the hill, are removed from the influence of decaying vegetable or animal matters."—ADDISON, 5.

The above extracts, which could not be conveniently abridged, will convey some local information relative to Hastings, and Malvern, as well as specimens of style—while it may be added that the publications of Mr. Addison, and of

Dr. Harwood well deserve, and especially that of the former, (who is obviously a candid, sensible, and practical man) an attentive perusal from those whom pleasure or sickness may call to these popular watering places.

The little work of Dr. Loudon, who is a remarkably well educated as well as talented physician, is interesting on account of the great celebrity which Leamington is rapidly acquiring, and which will soon render it a formidable rival to Cheltenham. The following extract will afford some idea of Dr. L.'s style.

"The sensible properties of the waters are the following. When drawn from the pumps, they are sparkling and transparent, and make a slight buzzing noise from the escape of their gaseous contents. The odour of the purely saline water, when newly taken from the fount, is slightly pungent; that of the sulphureous, somewhat like rotten eggs; and the chalybeate, inky. Their taste is either simply saline, sulphureo-saline, or saline-chalybeate, according to the ingredients of the spring. In winter the temperature is generally 44° of Fahrenheit's scale, and in summer about 10 degrees higher: thereby showing that they are under atmospheric influence. Exposed to the air, they continue for a short time to give out bubbles of gas, and soon become vapid. From their saline ingredients, however, they are less liable to freeze than simple water; and several of them, for the same reason, preserve their properties for years. All of them have a trace of iron in their composition, as well as of sulphur; the former being pointed out by the ochre deposited in the reservoirs underneath the pipes from whence they are drawn; the latter indicated, even in the purest saline, by the smell they emit before the fall of rain.

"In what particular way the mineral waters of Leamington acquire their impregnations, is by no means known. Various borings in the fields around the town, distinctly prove that the sources of the wells are confined to the strata underneath the houses, and at a very short distance around them—perhaps not more than a quarter of a mile. The difference between the temperature of summer and winter, may be owing to the water descending from the surface to the deeper parts of the earth before it reaches the spot where the saline impregnations are acquired; and this opinion seems strengthened by the well-known fact, that the level of the mineral springs is higher than the level of the water contained in the wells employed for domestic uses. It has also been thought probable, that there exist, underneath the town of Leamington, beds of salt, or brine pits, such as those at Northwich, in Cheshire, and Droitwich, in Worcestershire; and that in passing over these deposits, the fresh water, in descending from the surface, acquires at least a part of its saline ingredients; during which time it becomes cooled to the temperature which has been already noticed: but the existence of these saline beds, or brine pits, is, however, purely hypothetical.

"That the mineral waters acquire their solid contents before they become charged with the gaseous, is inferred from the small quantity and few kinds of aerial substances which are usually found in common springs. To explain the mode in which the Leamington wells acquire their gaseous contents, it has been supposed that there exists also, near the salt beds, a layer, or number of layers, of vegetable matter, from whence the gases are extricated by the fluid which has been already considered, as charged with the saline particles, and that through these layers it passes; here it extricates and carries along with it its gaseous contents; and, advancing nearer the surface, again meets with a stratum of pyrites or sulphuret of iron, which is combined with the silica, and which, in digging Mr. Smart's well, has been found to exist under the town. On meeting with this substance, the hydrogen unites with the sulphur, and forms the hepatic gas, and the bi-silicate of iron is taken up in solution." 15.

Although various analyses have been made, from time to time, of the Leamington waters, yet the following will be looked upon as the surest test, being made by Professor Thomas Thompson, of Edinburgh.

CONTENTS OF AN IMPERIAL PINT OF EACH OF THE LEAMINGTON MINERAL WATERS.

| SPRINGS. | | GASES, IN CUBIC INCHES. | | | | SALTS, IN GRAINS. | | | | | | Total of the Salts in Grains. |
|---|---|---|---|---|---|---|---|---|---|---|---|---|
| | | Oxygen. | Azote. | Carbonic Acid. | Sulphuretted Hydrogen | Sulphate of Soda. | Chloride of Sodium. | Chloride of Calcium. | Chloride of Magnesium | Silica. | Peroxide of Iron. | |
| Lord Aylesford's | | ·075 | ·537 | 2·103 | none | 40·398 | 40·770 | 20·561 | 3·266 | none | a trace | 105·095 |
| Mr. Smith's | | ·045 | ·658 | 2·503 | none | 40·234 | 47·865 | 19·772 | 2·121 | none | a trace | 109·992 |
| Mr. Wise's | | ·088 | ·488 | 2·180 | none | 39·457 | 26·610 | 18·737 | 22·592 | none | a trace | 107·396 |
| Mr. Robbins' | | ·075 | ·558 | 2·356 | none | 28·619 | 35·350 | 23·511 | 8·468 | none | a trace | 95·948 |
| Mr. Reid's | Sulphureous | ·025 | ·425 | 3·156 | 1·144 | 28·065 | 25·605 | 15·777 | 9·695 | none | a trace | 79·142 |
| | Saline | ·025 | ·565 | 2·162 | none | 30·610 | 42·922 | 17·987 | 10·813 | 0·972 | 0·265 | 103·575 |
| Imperial Fount. | Chalybeate | ·075 | ·645 | 3·294 | none | 34·294 | 55·271 | 25·059 | 3·927 | 8·580 | 8·580 | 135·711 |
| | Saline | ·098 | ·763 | 3·156 | none | 34·435 | 14·534 | 17·570 | 26·050 | none | a trace | 92·589 |
| | Sulphureous | ·012 | ·612 | 3·531 | 1·142 | 31·112 | 7·301 | 39·305 | 19·494 | 3·620 | 0·530 | 101·362 |
| Royal Pump Room. | Sulphureous | ·064 | ·498 | 3·156 | 1·140 | 5·546 | 5·144 | 3·365 | 1·156 | none | a trace | 15·211 |
| | Saline | ·066 | ·588 | 2·950 | none | 32·744 | 67·782 | 20·902 | 12·363 | 1·045 | 0·956 | 135·792 |

We are informed by Dr. L. that the Waters of Leamington may be taken at all seasons of the year, with perfect safety—though the best is from the beginning of May till the end of October. There is no difference produced in the waters by season; but the Leamington physicians wisely choose that one which offers the greatest auxiliaries to the springs, in the way of air, exercise, amusement, &c. when these addenda are withdrawn, the most celebrated mineral waters in the world lose much of their efficacy.

Preparatory to a course of the Leamington Waters, some aperient medicines are indispensible, sometimes with local or general bleeding, according to the nature of the case.

"The preparatory steps being premised, a common pint of the water may be taken; one half in the morning, about seven o'clock, on an empty stomach; the other in about twenty minutes afterwards; walking exercise being used between the first and second part of the dose, and after both have been taken. For children of twelve years of age, one-half of this quantity will be sufficient; for those of six, one-fourth. Under the age of six, they should scarcely ever be employed.

"The effects of the waters, when taken internally, in aperient doses, are of three kinds. Either they produce an increased action of the kidneys, or bring on nausea, sickness, head-ache, flushings of the face, distention of the stomach, determination to the head, and other disagreeable symptoms; or, finally and most frequently, they act on the bowels without inducing any of these signs. In the first case, they require only to be increased in the dose, to produce the aperient effect; while, in the second case, their use is not prohibited by the unfavourable effects arising from their employment, unless the remedies resorted to for removing them should prove ineffectual. The second class of symptoms frequently supervene from a deranged state of the alimentary canal; and by a little attention to the digestive organs, and to the dissipation of the gases, may be avoided. When they pass off by the kidneys, their use may always be regarded as pretty safe, and their action as salutary.

"When the waters sit easily and lightly on the stomach, and subsequently an increased flow of spirts and appetite follow, a favourable result may generally be anticipated. When the aperient effects are experienced in a few hours, without tormina or relaxation of the bowels, or that general lassitude of the system which arises from the frequent use of the drastic purges, or even from the milder aperients, such as manna, cassia, or senna, they may be considered as agreeing with the constitution, and their continuance will most likely be ultimately productive of the happiest effects. The number of times they require to be taken must necessarily depend on the disease, the kind of water employed, the constitutional predisposition of the invalid, and other circumstances. In general, twice or thrice a week will suffice, and a month should be allowed for trial." 45.

With this extract we must close Dr. Loudon's work, which is sensibly, ably, and modestly written. Such a publication is almost indispensible to the invalid visiting the springs of Leamington.

